# Toxic Effects of Silica Nanoparticles on Zebrafish Embryos and Larvae

**DOI:** 10.1371/journal.pone.0074606

**Published:** 2013-09-18

**Authors:** Junchao Duan, Yongbo Yu, Huiqin Shi, Linwei Tian, Caixia Guo, Peili Huang, Xianqing Zhou, Shuangqing Peng, Zhiwei Sun

**Affiliations:** 1 School of Public Health, Capital Medical University, Beijing, P.R. China; 2 Beijing Key Laboratory of Environmental Toxicology, Capital Medical University, Beijing, P.R. China; 3 Institute of Disease Control and Prevention, Academy of Military Medical Sciences, Beijing, P.R. China; 4 School of Public Health and Primary Care, Chinese University of Hong Kong, Hong Kong, P.R. China; Dowling College, United States of America

## Abstract

Silica nanoparticles (SiNPs) have been widely used in biomedical and biotechnological applications. Environmental exposure to nanomaterials is inevitable as they become part of our daily life. Therefore, it is necessary to investigate the possible toxic effects of SiNPs exposure. In this study, zebrafish embryos were treated with SiNPs (25, 50, 100, 200 µg/mL) during 4–96 hours post fertilization (hpf). Mortality, hatching rate, malformation and whole-embryo cellular death were detected. We also measured the larval behavior to analyze whether SiNPs had adverse effects on larvae locomotor activity. The results showed that as the exposure dosages increasing, the hatching rate of zebrafish embryos was decreased while the mortality and cell death were increased. Exposure to SiNPs caused embryonic malformations, including pericardial edema, yolk sac edema, tail and head malformation. The larval behavior testing showed that the total swimming distance was decreased in a dose-dependent manner. The lower dose (25 and 50 µg/mL SiNPs) produced substantial hyperactivity while the higher doses (100 and 200 µg/mL SiNPs) elicited remarkably hypoactivity in dark periods. In summary, our data indicated that SiNPs caused embryonic developmental toxicity, resulted in persistent effects on larval behavior.

## Introduction

Silica nanoparticles (SiNPs) have been found extensive applications in biomedical and biotechnological fields, such as medical diagnostics, drug delivery, gene therapy, tracking and imaging in vivo [1], [2], [3], [4](Li, 2012 #1). SiNPs is in the top five of nanomaterials explicitly referenced in nanotech-based comsumer products [5]. The toxicity research of nanomaterials is getting great attention as the increasing exposure of nanomaterials on human and ecological environment [6], [7]. One of the challenges in the field of nanotechnology is environmental health and safety (EHS), which is focusing on the consideration of the properties of engineered nanomaterials (ENMs) that could pose hazards to the environment and human beings [8].

Currently, zebrafish is emerging as a correlative in vivo vertebrate model for nano EHS studies due to their lower husbandry cost, optical transparency and high degree of genomic homology to humans [9], [10]. The zebrafish model has been reported for assessing of a wide array of nanomaterials including metal or metal oxide nanoparticles, carbon-based nanomaterials and polymers [11], [12], [13]. However, most studies conducted the embryonic toxicity induced by nanomaterials rather than assessing the changes of larval behavior. Zebrafish is also a popular model for the study of nervous system development [14]. New-hatched, larval zebrafish have a rich behavioral response. By six days post fertilization (dpf), the larvae are mature swimmers with functioning sensory and motor systems allowing studies of locomotor, escape, goal-oriented, and optomotor responses [15]. Thus, it is necessary to perform larval behavior as well as embryonic toxicity as evaluating nanomaterials. Despite the increasing popularity of SiNPs in biological applications, there is still lack of in vitro and in vivo data for predictive and correlative SiNPs toxicity. So far, only a few studies investigated the assessment of SiNPs toxicity using zebrafish model [16], [17]. Therefore, more studies are needed to better understand the toxicity of SiNPs in both embryos and larvae of zebrafish.

To our best knowledge, this is the first study to illustrate the embryonic toxicity and the alteration of larvae locomotor activity after zebrafish embryos exposure to SiNPs for 4–96 hours post fertilization (hpf). Prior to undertaking in vitro toxicity experiments, the characterization of SiNPs, which is essential for nanotoxicity studies, was performed by transmission electron microscope (TEM) and dynamic light scattering (DLS) measurements. To investigate the toxic effects of zebrafish embryos induced by SiNPs, we conducted a sequence of assessments including embryonic mortality, hatching rate, malformation and whole-embryo cellular death. We also determined the total swimming distance for light-dark optomotor responses to analyze whether SiNPs exposure could alter locomotor activity in zebrafish larvae. Taken embryonic toxicity and larval behavior together as indicators of evaluating SiNPs toxicity, will be more beneficial and comprehensive for the nano EHS studies and safety evaluation.

## Materials and Methods

### Silica Nanoparticles Preparation and Characterization

SiNPs were prepared using the Stöber method [18]. Briefly, 2.5 mL of tetraethylorthosilicate (TEOS) (Sigma, USA) was added to premixed ethanol solution (50 mL) containing ammonia (2 mL) and water (1 mL). The reaction mixture was kept at 40°C for 12 h with continuous stirring (150 r/min). The resulting particles were isolated by centrifugation (12,000 r/min, 15 min) and washed three times with deionized water and then dispersed in 50 mL of deionized water. The size of SiNPs was performed by transmission electron microscope (TEM) (JEOL JEM2100, Japan), and the size distribution was measured using ImageJ software (National Institutes of Health, USA). The hydrodynamic sizes and zeta potential of SiNPs were examined by Dynamic light scattering (DSL) technique using Zetasizer (Malvern Nano-ZS90, Britain). Suspensions of SiNPs were dispersed by sonicator (160 W, 20 kHz, 5 min) (Bioruptor UDC-200, Belgium) before addition to culture medium in order to minimize their aggregation. The purity of SiNPs was detected by Inductively Coupled Plasma- Atomic Emission Spetrometer (ICP-AES, ARL 3520 USA).

### Zebrafish Husbandry and Exposure to Silica Nanoparticles

Zebrafish of the AB strain (wild-type, wt) were raised on a circulating aquarium system in an environmentally controlled room (28°C, 80% humidity). The photoperiod was adjusted to a 14 h light/10 h dark cycle. The larval and adult zebrafish were fed with brine shrimp (hatched from eggs in 10 mL in 2 L salt water) daily. For experiments, fertilized eggs were collected and chosen under a stereomicroscope (Olympus SZX10, Tokyo, Japan) within 4 hours post fertilization (hpf). All embryos were derived from the same spawns of eggs for statistical comparison between control and treated groups. Healthy embryos were placed in 24-well culture plates (10 embryos in 2 ml solution/well). Each group had four replicate wells. Each experiment was replicated three times. At all stages, the developing embryos and larvae were maintained at 28°C in 30% Danieau’s Solution [58 mM NaCl, 0.7 mM KCl, 0.4 mM MgSO_4_, 0.6 mM Ca(NO_3_)_2_, 5 mM Hepes, pH 7.4]. Newly fertilized embryos were treated with SiNPs (25, 50, 100 and 200 µg/mL) for 4–96 hpf. For valid experiments, fertilized eggs were obtained only from spawns with a fertilization rate higher than 90%. In all experiments, dead embryos and larvae were removed from the 24-well plates every 12 h. We confirm that the Institutional Animal Care and Use Committee (IACUC) at the Academy of Military Medical Sciences of China have approved our study. All studies were carried out strictly according to the guidelines of the IACUC.

### Embryonic Toxicity

Zebrafish embryos exposed to SiNPs (25, 50, 100 and 200 µg/mL) for 4–96 hpf were measured for toxic effects of a continuing observation period. The SiNPs solutions were renewed and embryonic/larval mortality and hatching rate were evaluated every 24 h. The hatching rate is a ratio of hatching embryos to the reaming living embryos in each well. During the exposure period (4–96 hpf), the photographs of embryos malformation were captured under a stereomicroscope (Olympus SZX10, Tokyo, Japan) and the percentage of abnormal embryos was counted every 24 h. The photographs of embryos malformation were captured and differences were observed and noted.

### Cellular Death Assay

Cell death was detected in live embryos using acridine orange (AO) staining, a nucleic acid selective metachromatic dye that interacts with DNA and RNA by intercalation or electrostatic attractions [19]. AO stains cells with disturbed plasma membrane permeability so it preferentially stains necrotic or late apoptotic cells, whereas normal cells are non-permeable to AO. Embryos were exposed to SiNPs (25, 50, 100 and 200 µg/mL) for 4–28 hpf. Then embryos were rinsed three times with PBS and incubated in 5 µg/ml AO for 30 min in the dark at 28°C, followed by three times rinses in PBS. Stained embryos were examined using fluorescence microscopy (Olympus BX61, Tokyo, Japan). Whole-embryo fluorescence was measured and quantified using Volocity Demo 6.1.1 software (PerkinElmer, USA) [20].

### Larval Behavior Assay

Behavioral testing was performed at the 6 dpf time point (after the ending of SiNPs exposure) between 13∶30 and 16∶00 p.m. Larvae were cultured in 24-well plates at a density of one embryo per well. For all tests, the larvae were placed in 30% Danieau’s Solution without SiNPs. The visible light test was allowed larvae to acclimate to the well in light conditions for 20 min and then locomotor activities were recorded for the ensuing 3 min using a Noldus tracking device (Noldus Information Technology, Wageningen, Netherlands) and Media Cruiser recording software (Canopus Corporation, Kobe, Japan). Videos of larvae locomotor activities were assessed using EthoVision XT 7.0 software (Noldus Information Technology, Wageningen, Netherlands) to calculate total swimming distance (mm).

The light-dark test is conducted as the literatures and our previous study described [21], [22]. Briefly, the test consisted of acclimating larvae in the dark for 20 min, after which a cycle of 5 min in the dark, then 5 min in the light, was repeated over a course of 35 min. Data files were processed using EthoVision XT 7.0 software to calculate total swimming distance (cm) for each light or dark period.

### Statistical Analysis

Data were expressed as mean ± S.D. and significance was determined by using one-way analysis of variance (ANOVA) followed by Tukey’s test to compare the differences between groups. For the mortality and hatching rate, the two-way ANOVA was used with dosage and time. Differences were considered significant at *p*<0.05.

## Results

### Characterization of Silica Nanoparticles

**Figure 1:**
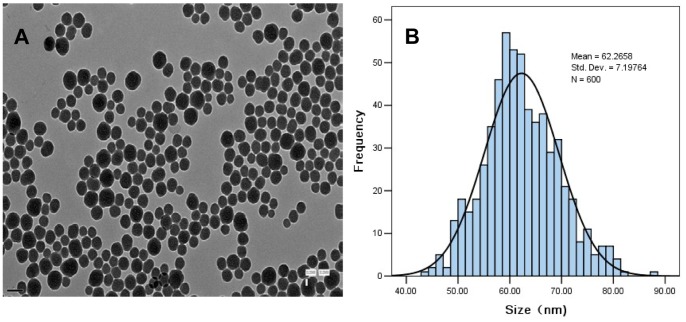
Characterization of silica nanoparticles. (A) Transmission electron microscopy image. (B) Size distribution. Silica nanoparticles exhibited good monodispersity and showed approximately normal distribution.

As shown in Figure 1A, the TEM images of SiNPs had a spherical shape with an average diameter of 62 nm. The size distribution was measured by ImageJ software showed approximately normal distribution (Figure 1B). The hydrodynamic sizes of SiNPs were measured in distilled water as stock media and in 30% Danieau’s Solution as culture media at different time point (Table 1). Our data showed SiNPs exhibited good monodispersity in 30% Danieau’s Solution. Zeta potentials provide quantitative information on the stability of the particles. SiNPs tested in our study had the absolute value of zeta potentials is higher than 30 mV. It is well documented that the particles are more likely to remain dispersed if the absolute value of zeta potential is higher than 30 mV [23]. Our results demonstrated that the SiNPs in culture medium possessed uniform shape along with relatively favorable dispersibility. The trace metal impurity levels in SiNPs are Ca 0.1400, Fe 0.00205, Mg 0.00325, Al 0.00078, Mn 0.00051, Cr 0.00090 (µg/10 mg SiO_2_), respectively. The purity of SiNPs was calculated higher than 99.9%.

**Table 1:** Hydrodynamic size and Zeta potential of silica nanoparticles in dispersion media.

	Distilled water		30% Danieau’s solution	
Time (h)	Diameter (nm)	Zeta potential (mV)	Diameter (nm)	Zeta potential (mV)
1	107.31±0.81	−38.10±0.46	107.03±0.83	−38.10±2.31
3	106.50±0.54	−39.13±5.26	106.83±0.64	−37.97±3.46
6	106.70±1.01	−41.43±3.29	107.21±0.61	−40.53±1.64
12	105.24±0.87	−44.10±1.30	105.60±0.79	−44.57±3.69
24	104.79±1.05	−46.33±3.13	104.93±0.61	−43.10±2.36

Data are expressed as means ± S.D. from three independent experiments.

### Mortality and Hatching Rate of Embryos Induced by Silica Nanoparticles

**Figure 2:**
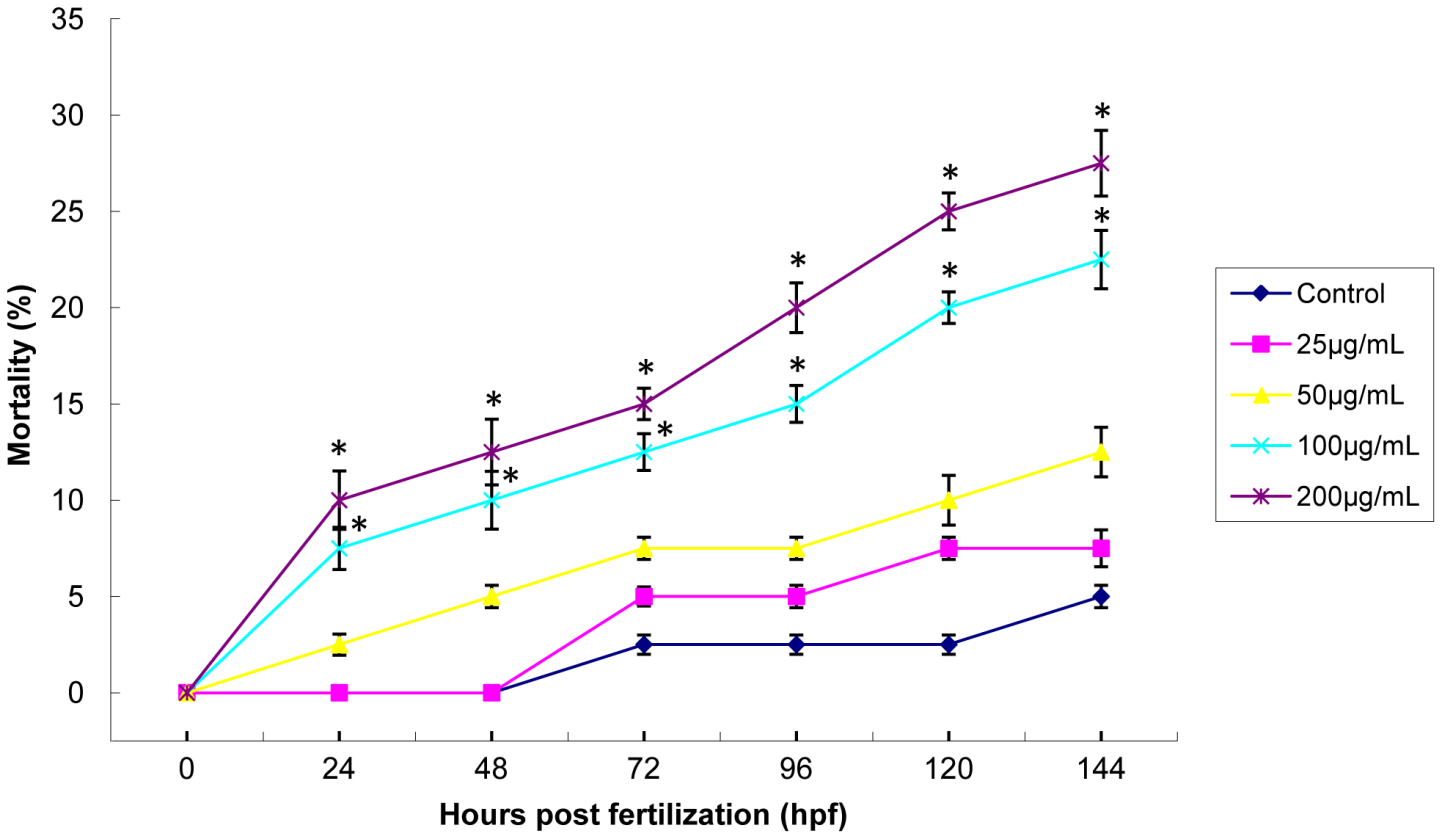
Mortality of zebrafish embryos exposed to silica nanoparticles. The mortality of embryos increased in dose- and time- dependent manner. Data are expressed as means ± S.D. from three independent experiments (*p<0.05).

**Figure 3:**
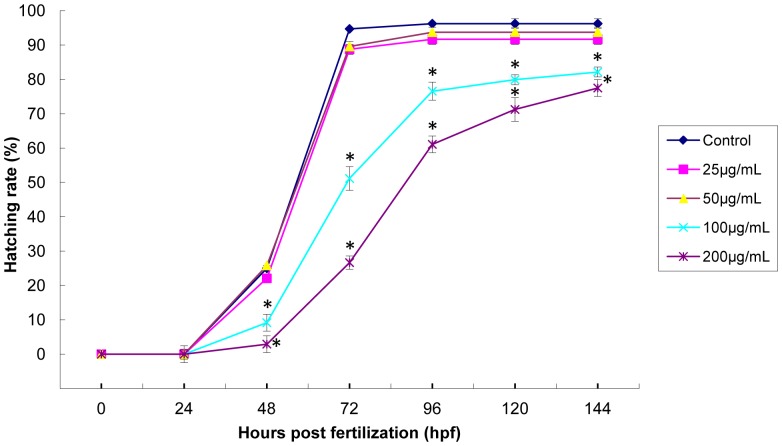
Hatching rate of zebrafish embryos induced by silica nanoparticles for 4–144 hpf. The results showed a strong inhibition of hatching rate after embryos exposed to silica nanoparticles. Data are expressed as means ± S.D. from three independent experiments (*p<0.05).

To evaluate the possible toxicity of zebrafish embryos exposure to SiNPs (25, 50, 100 and 200 µg/mL), we measured the mortality and hatching rate during a continuing observation period. As shown in Figure 2, at the lower concentration, there was no significant difference in mortality. With the dosages increasing, the mortality of 100 and 200 µg/mL treated groups increased significantly compared to that of control group. Normal embryos have a hatching period from 48 hpf to 72 hpf. Figure 3 showed a strong inhibition of hatching rate after embryos exposed to SiNPs. At 72 hpf, the hatching rate of 200 µg/mL group (26.67%) was much lower than that of control (94.72%). Our data showed that exposed to SiNPs caused the embryonic toxicity increased in a dose- and time- dependent manner.

### Malformation of Embryos Caused by Silica Nanoparticles

**Figure 4:**
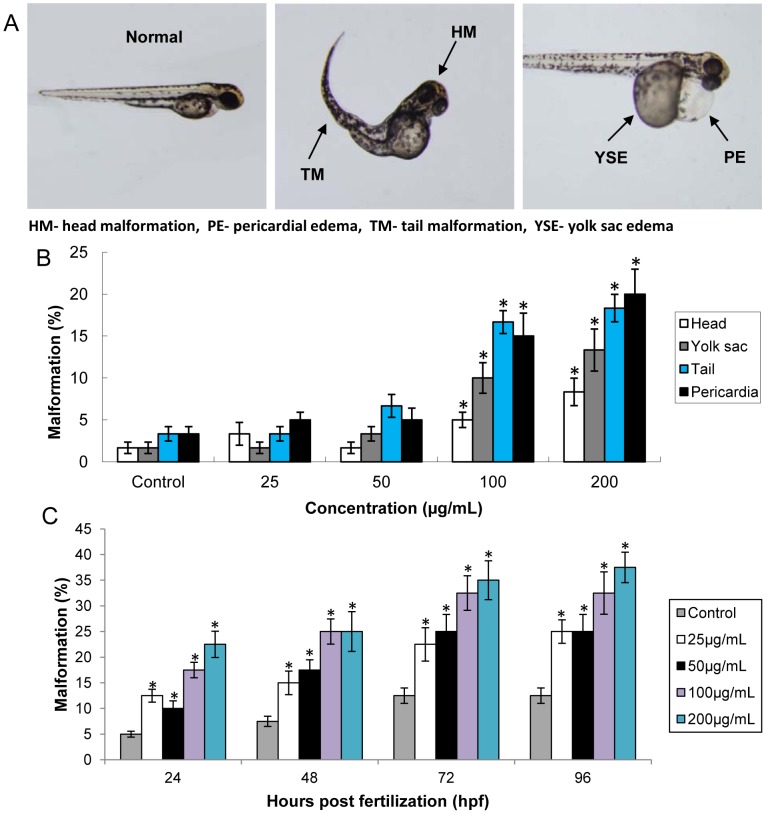
Malformation of zebrafish embryos exposed to silica nanoparticles. (A) Representative optical images of deformed zebrafish. (B) Pericardial and tail malformation were mainly typically malformation of embryos induced by silica nanoparticles. (C) Time-course variations of zebrafish embryos malformations induced by silica nanoparticles. Scale bar: 500 µm. Data are expressed as means ± S.D. from three independent experiments, n = 40 (*p<0.05).

As shown in Figure 4A, exposure to SiNPs caused embryos failed to development the normal morphology, the typically malformations including pericardial edema, yolk sac edema tail and head malformation. The incidence of embryonic morphological endpoints was observed as: Pericardia>Tail>Yolk sac>Head (Figure 4B). SiNPs induced embryos malformations in a time-course variations (Figure 4C). Our data showed that pericardial edema and tail malformation were mainly typically malformation of embryos induced by SiNPs.

### Embryonic Cell Death Induced by Silica Nanoparticles

**Figure 5:**
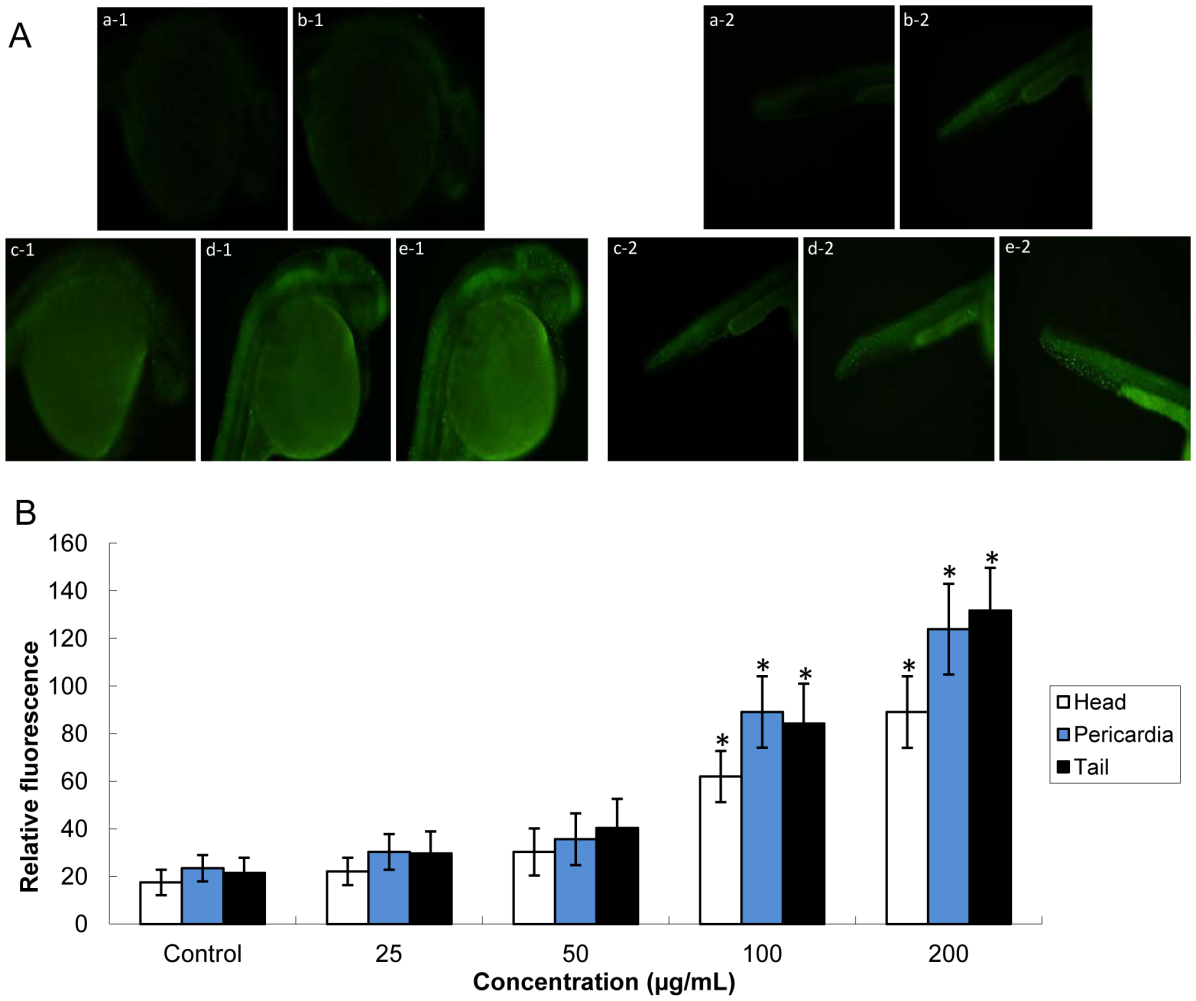
Cellular death was determined using acridine orange staining of silica nanoparticles-exposed embyos at 28 hpf, after a 24 h exposure. (A) Whole-embryo cell death images were detected by fluorescence microscope. a-1,a-2, Control group; b-1,b-2, 25 µg/mL treated group; c-1,c-2, 50 µg/mL treated group; d-1,d-2, 100 µg/mL treated group; e-1,e-2, 200 µg/mL treated group. (B) The relative fluorescence of cell death was detected as: Pericardia>Tail>Head. Scale bar: 500 µm. Data are expressed as means ± S.D. from three independent experiments, n = 40 (*p<0.05).

Cellular death assays were performed to determine whether exposure to SiNPs would lead to an increase in cellular death in specific cells or tissues, prior to the overt signs of toxicity shown in Figure 4. Embryos treated with SiNPs exhibited a dose-dependent increase in overall cellular death, significant at higher concentrations (100 and 200 µg/mL) (Figure 5). The distribution of cells death was mainly gathered in the head region, pericardia region and down the notochord to the region of tail. Our data showed that the relative fluorescence of cell death was detected as: Pericardia>Tail>Head. The whole-embryo fluorescence analysis was confirmed with the incidence of malformation results, indicated that pericardia and tail region were more sensitive to SiNPs.

### Alteration of Larval Locomotor Activity

**Figure 6:**
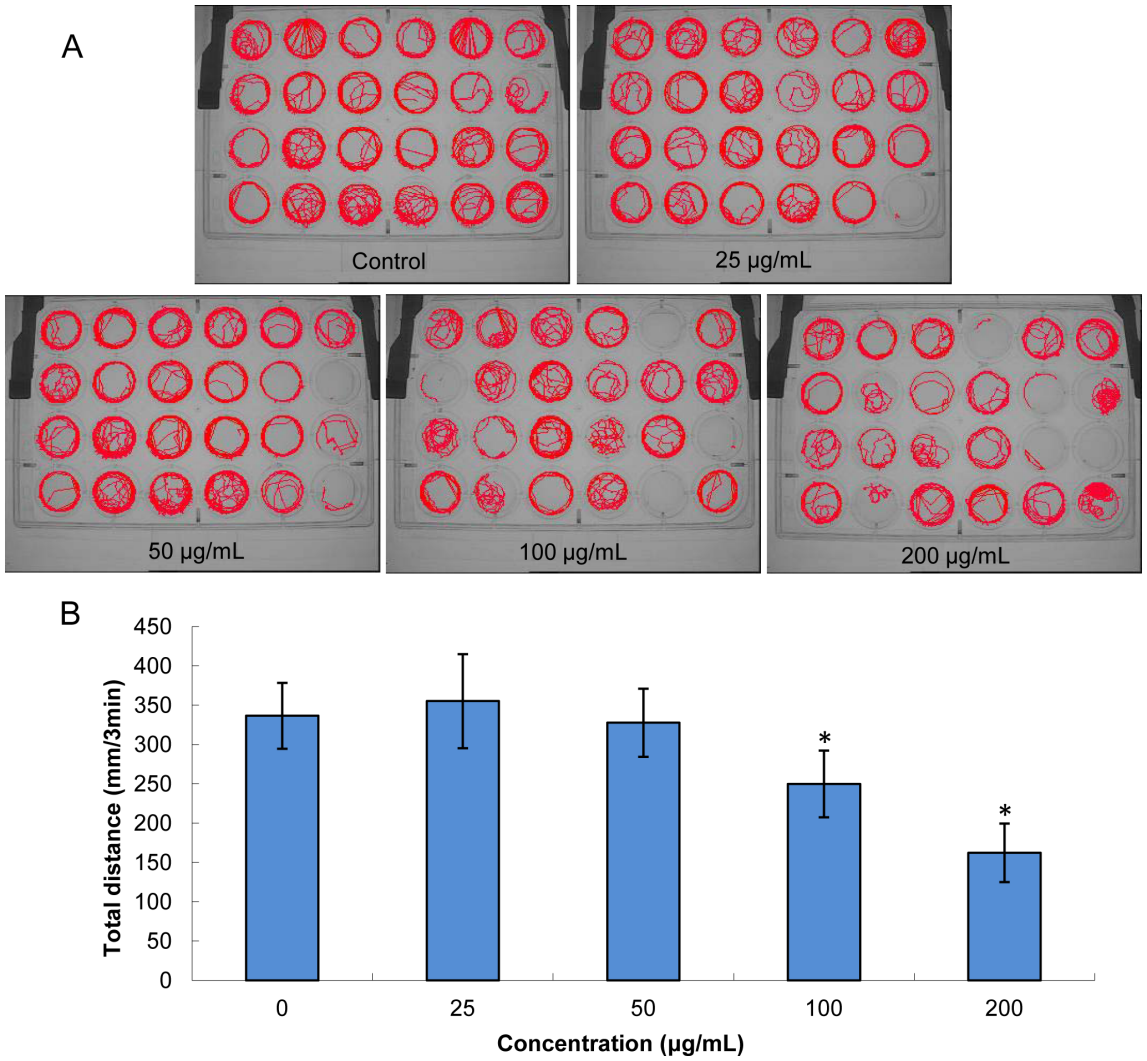
Persistent effects of silica nanoparticles on behavioral in larvae. (A) Tracking images of larval locomotor activity reduced gradually with the doses increasing. (B) Total swimming distance was decreased in a dose-dependent manner. Data are expressed as means ± S.D. from three independent experiments (*p<0.05).

**Figure 7:**
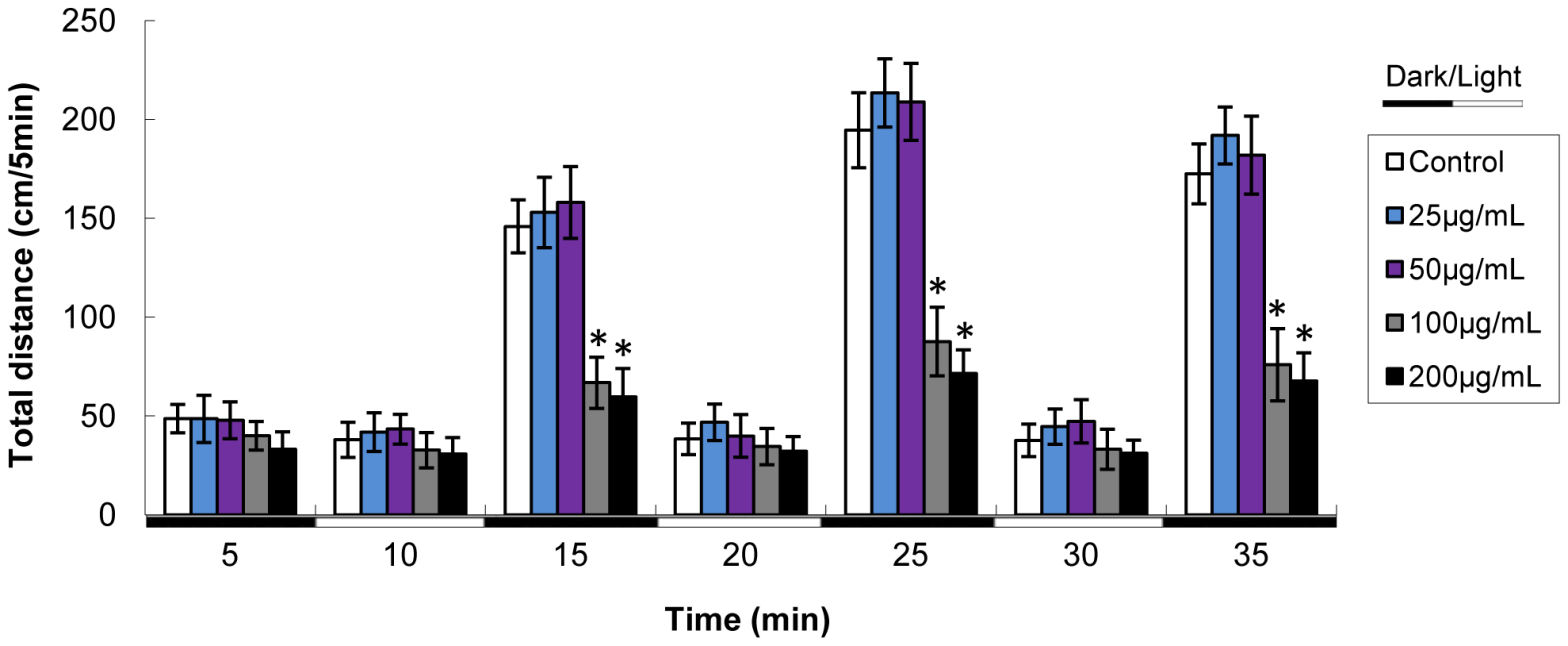
Locomotor activity of zebrafish larvae using the tracking sets during alternating periods of light and dark. White and black bars at the bottom denote light and dark periods, respectively. Data are expressed as means ± S.D. from three independent experiments, n = 24 (*p<0.05).

We determined the locomotor activity of larvae at 6 dpf time point (after the ending of SiNPs exposure period) to analyze whether SiNPs exposure could have a persistent effects on larval behavior. In the visible light test, we can obtain from the tracking images that the larval locomotor activity reduced gradually with the dosage increasing (Figure 6A). These tracking images were consistent with the total swimming distance which was decreased in a dose-dependent manner (Figure 6B). In the light-dark test, we measured the locomotor activity using the tracking sets during alternating periods of light and dark. As shown in Figure 7, larval zebrafish subjected to this test typically move more active during dark periods and less active during the light periods. The lower treated groups (25 and 50 µg/mL SiNPs) did produce substantial hyperactivity but without significant difference compared to that of control group. While the higher treated groups (100 and 200 µg/mL SiNPs) elicited significant hypoactivity, but only during the dark periods.

## Discussion

Environmental exposure to nanomaterials is inevitable as nanomaterials become part of our daily life. As a result, nanotoxicity research is gaining attention. In this study, we reported for the first time that zebrafish exposure to SiNPs (25, 50, 100, 200 µg/mL) during 4–96 hpf embryonic period resulted in a persistent effects on larval behavior. Our findings demonstrated that zebrafish exposure to SiNPs led to embryonic developmental toxicity and altered larval locomotor activity, which may provide more persuasive experimental data for the nano EHS studies and safety evaluation.

It is recommended that prior to the toxicity research nanomaterials should be detailed characterized in order to gain a better explanation for experiments [24], [25]. In this study, TEM and DSL were utilized to test the features of SiNPs. TEM could determine the morphous and original diameter of the particles (Figure 1). DLS mainly reflects the hydrodynamic size in dispersion media (Table 1). SiNPs used in our research are spherical and the hydrodynamic size was measured in distilled water as stock media as well as in 30% Danieau’s Solution as exposure media in different time points. The data showed that owing to the Van der Waals force and hydrophobic interaction with surrounding media the hydrodynamic size is generally larger than original [26]. In addition, SiNPs exhibited very good monodispersity in the experimental process since the absolute value of zeta potential is higher than 30 mV [23]. The reasons for silica nanoparticles exhibited the high zeta potential values could be as follows: The hydrophilicity of silica material increases with the number of silanols, or silicon-bonded hydroxyl groups, capable of forming hydrogen bonds with physical water molecules [27]. The reported concentration of hydroxyl groups per square nanometer on the surface of amorphous silica ranges from 4 to 5 OH/nm^2^, which has generally contain a higher concentration of surface hydroxyl groups [28]. Our results showed that the SiNPs to be tested possess uniform shape and structure along with relatively favourable dispersibility which are conducive to the following nanotoxicity research.

Despite the increasing popularity of nanomaterials in biological applications, there is still lack of in vitro and in vivo data for predictive and correlative nanomaterials toxicity. It is a fairly simple and cost-effective process to initially screen nanomaterial toxicity by in vitro cell culture; unfortunately, it is nearly impossible to imitate a complimentary in vivo system. Small mammalian models remain the putative method to assess the possible toxicities and biodistribution of nanomaterials in humans. However, establishing mammalian models is often expensive and time-consuming. Instead, the millimeter-sized zebrafish are proving to be a quick and facile model to conservatively assess toxicity of nanomaterials. Utility of zebrafish for biotoxicity screens is largely based upon the close homology with the human genome. In addition, zebrafish and mammals demonstrate a similar physiologic response when introduced to xenobiotics. Furthermore, the biology, optical clarity and high-throughput screening of zebrafish embryos allows testing at all stages of embryonic development [10]. It was well documented that earlier developmental embryos are more sensitive to external substances than larval or adult zebrafish [29]. Therefore, the embryonic period (4–96 hpf) was chosen as administration time to study the possible toxicity of SiNPs. In the present study, our data showed that exposure to SiNPs caused the increasing of mortality and the inhibition of hatching rate in a dose- and time- dependent manner (Figure 2 and Figure 3). Consistent with our findings, decreasing survival and hatching rate of zebrafish embryos have been observed in various nanomaterials [30], [31], [32]. The inhibition of hatching rate led to a direct delay of embryos development. From this study, we observed several types of malformation in embryos incubating with SiNPs (25, 50, 100 and 200 µg/mL), including pericardial edema, yolk sac edema, tail and head malformation (Figure 4A). The results in Figure 4B showed that the pericardial edema and tail malformation occurred as common malformations observed in embryos exposed to SiNPs. Similar with our results, Xu and coworkers reported that serious malformations of pericardial edema and yolk sac edema were found in embryos treated with titanium dioxide nanoparticles [33]. While fin fold and tail abnormalities were observed as the most common ones when embryos incubating with sliver nanoparticles [34]. Thus, we could also confirm that different nanomaterials led to different types of malformations in zebrafish embryos.

To better understand the overt signs of malformation reported in Figure 4, whole-embryo cellular death assays were detected by AO staining (Figure 5A). Figure 5B showed that exposure to SiNPs for 24 h led to an increase in cellular death in specific region: Pericardia>Tail>Head. AO binds all cells undergoing cellular death, both necrosis and apoptosis [19], [35]. In addition, the mechanism of cellular death induction is an important concern since its correlation with overall tissue damage: necrosis tends to cause extensive tissue damage resulting in an inflammatory response in vivo; while apoptosis does not cause tissue damage since macrophages effectively remove apoptotic signaling cells [13]. AO staining for cellular death, as a sensitive endpoint indicator, was consistent with the overt signs of embryos malformation images.

Currently, most studies have focused on understanding how acute exposure of nanomaterials induced toxic effects using both in vitro and in vivo models [36], [37], [38]. While these studies are critical and provided informative data, there are other areas of potential consideration regarding nanomaterials that have yet to be explored, including persistent effects following acute exposure. Our study conducted that embryos exposed to SiNPs only during embryonic period (4–96 hpf), then raised in culture medium to determine the persistent effects on larval behavior. The larval zebrafish is emerging as a promising high-throughput model for neurobehavioral research due to its well-characterized genome, robust behavioral responses, and physiological similarity to human [39], [40].

In the present study, our data showed that the total distance was decreased in a dose-dependent manner in the visible light test (Figure 6). We also manipulated a light and tap stimulus test due to their significant effects on larval locomotor activity [41], [42]. As shown in the light-dark test (Figure 7), alternating light and dark periods produced a consistent pattern of locomotor activity: in visible light, the larvae first ceased movement, and then slowly increased activity over the ten-minute period, when darkness was imposed, the larval zebrafish rapidly and markedly increased activity, which then slowly abated with time. Also, larvae were characteristically more active in the dark periods that followed light periods than initial dark periods. The lower dose (25 and 50 µg/mL SiNPs) caused substantial hyperactivity in larval zebrafish, but without significant difference compared to control group; while the higher doses (100 and 200 µg/mL SiNPs) produced significantly hypoactivity during dark periods. It was interesting to note, the larval zebrafish of the higher concentration groups showed a lag response in light periods. We found that the persistent effects of SiNPs were altering locomotor activity in larval zebrafish. Our data showed that larvae treated with SiNPs exhibited an “inverted U”-shaped dose-response curve in the dark periods. Several studies reported that the patterns of neuroactive drugs using zebrafish model were similar to those obtained in rodent model [43], [44]. However, while altered larval behavior was suggested as an impact on nervous system development, it did not exclude the possibility that the SiNPs was affected on other target organs, such as the eyes (e.g., ability to detect light) or neuromuscular system (e.g., ability to swim). Further investigations will be required to determine the extent to which the nervous system or other target organs contribute to the observed behavioral phenotypes.

It was worth mentioning that the toxicity induced by SiNPs was related to the embryos chorion. The chorion has pores that are important for oxygen/carbon dioxide, nutrients and excretion product transport to and from the embryo, respectively. The chorion is an acellular envelope made of three intercrossing layers, which allows materials to pass through to the embryo via passive diffusion [45]. The chorion possess pore canals is to be approximately 0.5–0.7 µm in diameter, meaning that the size of chorion pore canals are larger than the common size of nanoparticles [46]. However, some studies have reported that the nano-SiO_2_ is mainly adsorbed on the out surface of chorion, which might affect the nutrient absorption and vitamin synthesis [16], [17]. In the present study, our data showed that exposure to silica nanoparticles caused the inhibition of hatching rate, which led to a direct delay of embryos development. Similar to our findings, delayed hatching observed with carbon nanotubes or nano-ZnO were regarded as an indirect effect of blocking O_2_-exchange [47], [48]. It is still unknown whether the nanoparticles possess specific or non-specific interactions with the chorion. Therefore, more studies are needed to clarify the interactions between the chorion and the nanoparticles.

## Conclusions

In summary, the present study demonstrates that SiNPs cause developmental embryonic toxicity, resulted in persistent effects on larval behavior. Thus, our findings suggest that exposure to SiNPs could be a potential hazardous factor for environmental exposure, more studies of relation between SiNPs exposure, adverse effects and biological mechanisms are needed for the EHS studies and safety evaluation of SiNPs.

## References

[1] Li Z, Barnes JC, Bosoy A, Stoddart JF, Zink JI (2012) Mesoporous silica nanoparticles in biomedical applications. Chem Soc Rev 41: 2590–2605. 22216418 10.1039/c1cs15246g

[2] Barandeh F, Nguyen PL, Kumar R, Iacobucci GJ, Kuznicki ML, et al (2012) Organically modified silica nanoparticles are biocompatible and can be targeted to neurons in vivo. PLoS One 7: e29424. 22238611 10.1371/journal.pone.0029424 PMC3250438

[3] Lee JE, Lee N, Kim T, Kim J, Hyeon T (2011) Multifunctional mesoporous silica nanocomposite nanoparticles for theranostic applications. Acc Chem Res 44: 893–902. 21848274 10.1021/ar2000259

[4] Sharif F, Porta F, Meijer AH, Kros A, Richardson MK (2012) Mesoporous silica nanoparticles as a compound delivery system in zebrafish embryos. Int J Nanomedicine 7: 1875–1890. 22605936 10.2147/IJN.S26547 PMC3352692

[5] Park MV, Verharen HW, Zwart E, Hernandez LG, van Benthem J, et al (2011) Genotoxicity evaluation of amorphous silica nanoparticles of different sizes using the micronucleus and the plasmid lacZ gene mutation assay. Nanotoxicology 5: 168–181. 20735203 10.3109/17435390.2010.506016

[6] Ostroumov SA, Kotelevtsev SV (2011) Toxicology of nanomaterials and environment. Ecologica 18: 3–10.

[7] Ray PC, Yu H, Fu PP (2009) Toxicity and environmental risks of nanomaterials: challenges and future needs. J Environ Sci Health C Environ Carcinog Ecotoxicol Rev 27: 1–35. 19204862 10.1080/10590500802708267 PMC2844666

[8] Thomas CR, George S, Horst AM, Ji Z, Miller RJ, et al (2011) Nanomaterials in the environment: from materials to high-throughput screening to organisms. ACS Nano 5: 13–20. 21261306 10.1021/nn1034857

[9] Lin S, Zhao Y, Nel AE, Lin S (2013) Zebrafish: An In Vivo Model for Nano EHS Studies. Small 9: 1608–1618. 23208995 10.1002/smll.201202115 PMC4070293

[10] Fako VE, Furgeson DY (2009) Zebrafish as a correlative and predictive model for assessing biomaterial nanotoxicity. Adv Drug Deliv Rev 61: 478–486. 19389433 10.1016/j.addr.2009.03.008

[11] Heiden TC, Dengler E, Kao WJ, Heideman W, Peterson RE (2007) Developmental toxicity of low generation PAMAM dendrimers in zebrafish. Toxicol Appl Pharmacol 225: 70–79. 17764713 10.1016/j.taap.2007.07.009 PMC6886473

[12] Lin S, Zhao Y, Xia T, Meng H, Ji Z, et al (2011) High content screening in zebrafish speeds up hazard ranking of transition metal oxide nanoparticles. ACS Nano 5: 7284–7295. 21851096 10.1021/nn202116p PMC4136441

[13] Usenko CY, Harper SL, Tanguay RL (2007) In vivo evaluation of carbon fullerene toxicity using embryonic zebrafish. Carbon N Y 45: 1891–1898. 18670586 10.1016/j.carbon.2007.04.021 PMC2186061

[14] Linney E, Upchurch L, Donerly S (2004) Zebrafish as a neurotoxicological model. Neurotoxicol Teratol 26: 709–718. 15451034 10.1016/j.ntt.2004.06.015

[15] Drapeau P, Saint-Amant L, Buss RR, Chong M, McDearmid JR, et al (2002) Development of the locomotor network in zebrafish. Prog Neurobiol 68: 85–111. 12450489 10.1016/s0301-0082(02)00075-8

[16] Fent K, Weisbrod CJ, Wirth-Heller A, Pieles U (2010) Assessment of uptake and toxicity of fluorescent silica nanoparticles in zebrafish (Danio rerio) early life stages. Aquat Toxicol 100: 218–228. 20303188 10.1016/j.aquatox.2010.02.019

[17] Xu Z, Zhang Y-L, Song C, Wu L-L, Gao H-W (2012) Interactions of Hydroxyapatite with Proteins and Its Toxicological Effect to Zebrafish Embryos Development, PLoS One. 7: e32818. 10.1371/journal.pone.0032818 PMC3324474 22509249

[18] Duan JC, Yu YB, Li Y, Yu Y, Li YB, et al (2013) Toxic effect of silica nanoparticles on endothelial cells through DNA damage response via Chk1-dependent G2/M checkpoint. PLoS One 8: e62087. 23620807 10.1371/journal.pone.0062087 PMC3631220

[19] Hu YL, Qi W, Han F, Shao JZ, Gao JQ (2011) Toxicity evaluation of biodegradable chitosan nanoparticles using a zebrafish embryo model. Int J Nanomedicine 6: 3351–3359. 22267920 10.2147/IJN.S25853 PMC3260029

[20] Duan JC, Yu YB, Li Y, Yu Y, Sun ZW (2013) Cardiovascular toxicity evaluation of silica nanoparticles in endothelial cells and zebrafish model. Biomaterials 34: 5853–5862. 23663927 10.1016/j.biomaterials.2013.04.032

[21] Truong L, Saili KS, Miller JM, Hutchison JE, Tanguay RL (2012) Persistent adult zebrafish behavioral deficits results from acute embryonic exposure to gold nanoparticles. Comp Biochem Physiol C Toxicol Pharmacol 155: 269–274. 21946249 10.1016/j.cbpc.2011.09.006 PMC3255321

[22] Duan JC, Yu YB, Li Y, Yu Y, Li YB, et al (2013) Developmental toxicity of CdTe QDs in zebrafish embryos and larvae. Journal of Nanoparticle Research 15: 1700.

[23] Jiang J, Oberdörster G, Biswas P (2009) Characterization of size, surface charge, and agglomeration state of nanoparticle dispersions for toxicological studies. Journal of Nanoparticle Research 11: 77–89.

[24] Balbus JM, Maynard AD, Colvin VL, Castranova V, Daston GP, et al (2007) Meeting report: hazard assessment for nanoparticles–report from an interdisciplinary workshop. Environ Health Perspect 115: 1654–1659. 18007999 10.1289/ehp.10327 PMC2072837

[25] Li Y, Sun L, Jin M, Du Z, Liu X, et al (2011) Size-dependent cytotoxicity of amorphous silica nanoparticles in human hepatoma HepG2 cells. Toxicol In Vitro 25: 1343–1352. 21575712 10.1016/j.tiv.2011.05.003

[26] Skebo JE, Grabinski CM, Schrand AM, Schlager JJ, Hussain SM (2007) Assessment of metal nanoparticle agglomeration, uptake, and interaction using high-illuminating system. Int J Toxicol 26: 135–141. 17454253 10.1080/10915810701226248

[27] Napierska D, Thomassen LC, Lison D, Martens JA, Hoet PH (2010) The nanosilica hazard: another variable entity. Part Fibre Toxicol 7: 39. 21126379 10.1186/1743-8977-7-39 PMC3014868

[28] Panessa-Warren BJ, Warrren JB, Maye MM, Schiffer W (2009) Nanoparticle Interactions with Living Systems: In Vivo and In Vitro Biocompatibility. In Nanoparticles and Nanodevices in Biological Applications 4: 1–45.

[29] Yan H, Teh C, Sreejith S, Zhu L, Kwok A, et al (2012) Functional mesoporous silica nanoparticles for photothermal-controlled drug delivery in vivo. Angew Chem Int Ed Engl 51: 8373–8377. 22777795 10.1002/anie.201203993

[30] Bar-Ilan O, Louis KM, Yang SP, Pedersen JA, Hamers RJ, et al (2012) Titanium dioxide nanoparticles produce phototoxicity in the developing zebrafish. Nanotoxicology 6: 670–679. 21830861 10.3109/17435390.2011.604438

[31] Zhu X, Zhu L, Duan Z, Qi R, Li Y, et al (2008) Comparative toxicity of several metal oxide nanoparticle aqueous suspensions to Zebrafish (Danio rerio) early developmental stage. J Environ Sci Health A Tox Hazard Subst Environ Eng 43: 278–284. 18205059 10.1080/10934520701792779

[32] Lee KJ, Nallathamby PD, Browning LM, Desai T, Cherukuri PK, et al (2012) Single nanoparticle spectroscopy for real-time in vivo quantitative analysis of transport and toxicity of single nanoparticles in single embryos. Analyst 137: 2973–2986. 22563577 10.1039/c2an35293a

[33] Xu Z, Zhang YL, Song C, Wu LL, Gao HW (2012) Interactions of hydroxyapatite with proteins and its toxicological effect to zebrafish embryos development. PLoS One 7: e32818. 22509249 10.1371/journal.pone.0032818 PMC3324474

[34] Lee KJ, Browning LM, Nallathamby PD, Desai T, Cherukuri PK, et al (2012) In vivo quantitative study of sized-dependent transport and toxicity of single silver nanoparticles using zebrafish embryos. Chem Res Toxicol 25: 1029–1046. 22486336 10.1021/tx300021u PMC3518489

[35] Lam KH, Alex D, Lam IK, Tsui SK, Yang ZF, et al (2011) Nobiletin, a polymethoxylated flavonoid from citrus, shows anti-angiogenic activity in a zebrafish in vivo model and HUVEC in vitro model. J Cell Biochem 112: 3313–3321. 21748787 10.1002/jcb.23257

[36] Zhu ZJ, Carboni R, Quercio MJ Jr, Yan B, Miranda OR, et al (2010) Surface properties dictate uptake, distribution, excretion, and toxicity of nanoparticles in fish. Small 6: 2261–2265. 20842664 10.1002/smll.201000989 PMC3024600

[37] Powers CM, Badireddy AR, Ryde IT, Seidler FJ, Slotkin TA (2011) Silver nanoparticles compromise neurodevelopment in PC12 cells: critical contributions of silver ion, particle size, coating, and composition. Environ Health Perspect 119: 37–44. 20840908 10.1289/ehp.1002337 PMC3018497

[38] Gao X, Yin S, Tang M, Chen J, Yang Z, et al (2011) Effects of developmental exposure to TiO2 nanoparticles on synaptic plasticity in hippocampal dentate gyrus area: an in vivo study in anesthetized rats. Biol Trace Elem Res 143: 1616–1628. 21331565 10.1007/s12011-011-8990-4

[39] Lieschke GJ, Currie PD (2007) Animal models of human disease: zebrafish swim into view. Nat Rev Genet 8: 353–367. 17440532 10.1038/nrg2091

[40] Riehl R, Kyzar E, Allain A, Green J, Hook M, et al (2011) Behavioral and physiological effects of acute ketamine exposure in adult zebrafish. Neurotoxicol Teratol 33: 658–667. 21683787 10.1016/j.ntt.2011.05.011

[41] MacPhail RC, Brooks J, Hunter DL, Padnos B, Irons TD, et al (2009) Locomotion in larval zebrafish: Influence of time of day, lighting and ethanol. Neurotoxicology 30: 52–58. 18952124 10.1016/j.neuro.2008.09.011

[42] Prober DA, Rihel J, Onah AA, Sung RJ, Schier AF (2006) Hypocretin/orexin overexpression induces an insomnia-like phenotype in zebrafish. J Neurosci 26: 13400–13410. 17182791 10.1523/JNEUROSCI.4332-06.2006 PMC6675014

[43] Irons TD, MacPhail RC, Hunter DL, Padilla S (2010) Acute neuroactive drug exposures alter locomotor activity in larval zebrafish. Neurotoxicol Teratol 32: 84–90. 19465114 10.1016/j.ntt.2009.04.066

[44] Burgess HA, Granato M (2007) Modulation of locomotor activity in larval zebrafish during light adaptation. J Exp Biol 210: 2526–2539. 17601957 10.1242/jeb.003939

[45] Berghmans S, Butler P, Goldsmith P, Waldron G, Gardner I, et al (2008) Zebrafish based assays for the assessment of cardiac, visual and gut function–potential safety screens for early drug discovery. J Pharmacol Toxicol Methods 58: 59–68. 18585469 10.1016/j.vascn.2008.05.130

[46] Lee KJ, Nallathamby PD, Browning LM, Osgood CJ, Xu XH (2007) In vivo imaging of transport and biocompatibility of single silver nanoparticles in early development of zebrafish embryos. ACS nano 1: 133–143. 19122772 10.1021/nn700048y PMC2613370

[47] Cheng J, Flahaut E, Cheng SH (2007) Effect of carbon nanotubes on developing zebrafish (Danio rerio) embryos. Environ Toxicol Chem 26: 708–716. 17447555 10.1897/06-272r.1

[48] Bai W, Zhang Z, Tian W, He X, Ma Y, et al (2010) Toxicity of zinc oxide nanoparticles to zebrafish embryo: a physicochemical study of toxicity mechanism, Nanoparticle Res. 12: 1645–1654.

